# Effect of coherent breathing on mental health and wellbeing: a randomised placebo-controlled trial

**DOI:** 10.1038/s41598-023-49279-8

**Published:** 2023-12-13

**Authors:** Guy W. Fincham, Clara Strauss, Kate Cavanagh

**Affiliations:** 1 Department of Psychology, University of Sussex, Brighton, UK; 2 Brighton & Sussex Medical School, University of Sussex, Brighton, UK; 3 Research & Development Department, Sussex Partnership NHS Foundation Trust, Brighton, UK

**Keywords:** Quality of life, Psychology, Human behaviour

## Abstract

Breathwork may offer simple tools for stress resilience. We conducted the largest parallel randomised-controlled trial on breathwork to date (NCT05676658) wherein 400 participants on the research platform Prolific were randomised, in blocks of 2 via remote software, to coherent breathing at ~ 5.5 breaths/min or a matched attention-placebo at 12 breaths/min, for ~ 10 min/day over 4 weeks. Participants were blinded to their allocated interventions, both of which were paced with equal inhalation:exhalation ratios. There were no differences on credibility and expectancy of benefit between conditions. At the primary timepoint post-intervention for the primary outcome subjective stress, there was no significant group by time interaction (*F*(1,377) = 0.089, *p* = 0.765, *η*_*p*_^2^ < 0.001) nor main effect of group (*F* = 0.002, *p* = 0.961, *η*_*p*_^2^ < 0.001), however there was a significant main effect of time (*F* = 72.1, *p* < 0.001, *η*_*p*_^2^ = 0.161). Similar results were found at 1-month follow-up for stress and for secondary outcomes of anxiety, depression and wellbeing. There were overall improvements on these mental health and wellbeing outcomes from baseline to post-intervention and follow-up across both groups, yet the magnitude of this improvement was not different between arms. Accordingly, we found no measurable effect of coherent breathing over and above a well-designed breathwork placebo at improving mental health and wellbeing. Methodological considerations and recommendations for robust future research are discussed. Funder: Sasakawa Young Leaders Fellowship Fund, Tokyo, Japan.

## Introduction

### Background

Breathwork may be defined as deliberate control of breath rhythm and pattern, usually with the aim of increasing focus and relaxation and/or with the aim of influencing emotional, mental and physical health^1,2^. Since ancient times myriad forms of breathwork have emerged independently around the globe via teachings by shamans, martial artists, tribal and religious leaders, including medieval orthodox Russian Christian monks, Hawaiian kahunas, Buddhist meditators, Indian yogis and Chinese Qigong practitioners^3^. The emergence of such techniques from various cultures and traditions all around the world have roots going back ~ 10,000 years, and perhaps the most well-known body of practices are derived from pranayama, one of the classical limbs of Yoga, which began receiving global scientific examination nearly 60 years ago^3–5^.

Fast forward to the present and breathwork has transformed and extended its reach to use and teaching by modern-day psychedelic groups, medical professionals, elite performers, military, along with practitioners of health and wellness more broadly. Public interest in breathwork has exploded due to its therapeutic potential, but it is essential such excitement is grounded in a robust evidence-base^6^.

Slow-paced breathwork has received most research attention thus far, and its physiological effects have been detailed extensively^7,8^. There is also emerging scientific interest in the psychological effects of these practices, the most robustly designed studies of which are synthesised in our recent meta-analysis on breathwork and mental health in randomised-controlled trials (RCTs)^6^. We found significant small-medium effects of breathing practices (mainly slow-paced) on subjective stress, anxiety and depressive symptoms compared to non-breathwork controls, however most studies displayed moderate risk of bias thereby clouding interpretation of such positive findings. More robust studies are needed to increase confidence in the effects of breathwork.

There is one particular rhythm of breathing (5–6 breath cycles per minute—bcpm—with no deliberate pauses) that is widely found in religions and cultures around the world (in Yoga, Qigong, prayer and mantra, along with meditation) and has been researched in scientific practice^3,9–11^. Such rhythmic breathwork is commonly referred to as coherent breathing (CB), since it is a breathing pace proposed to lead to coherence or synchronicity of respiration with cardiovascular functions, which may lead to improvements in stress, anxiety and depressive symptoms^12^. There may also be beneficial effects of slow-paced breathing on sleep outcomes^13,14^. However, despite over two decades of research, there are no well-controlled studies exploring such breathwork's effects on stress, mental health, sleep and wellbeing. Accordingly, nuanced interpretation of CBs positive effects is required paired with more robust study design for breathwork research in general^8,12^.

Research to date examining the efficacy of CB in improving mental health lacks internal validity as control conditions do not allow effects to be attributed to CB with confidence^15^. A well-designed, equally plausible, active attention-placebo would allow us to tease apart and establish any specific effects that may be accountable to CB per se (i.e., above and beyond intention, belief, attention, expectation and time commitment). Furthermore, degree of change on mental health outcomes will likely fluctuate as a function of the credibility and quality of an attention-placebo control^15^. This has proved to be a challenging area for clinical research owing to the complexity of developing behavioural placebos that yield as great a level of expectancy for change as the active intervention^15^. A recent review of the literature on slow breathwork at ~ 6 bcpm states that a more comprehensive use of placebos such as paced respiration at a natural frequency or interventions that are neutral in nature, would assist in separating effects of slow breathwork from those of purely distraction or awareness of respiration^12^.

### Objectives

Here, we planned to evaluate the effects of CB on stress, mental health, sleep and wellbeing outcomes in the (to the best of our knowledge) first robust RCT, comparing CB (at ~ 5.5 bcpm) to a well-designed placebo (12 bcpm with equal inhale:exhale ratios and no pauses). 12 bcpm is the lower bound of average resting respiration rate in adults. The theoretical rationale for our study was identifying whether CB exerts specific effects in a well-designed RCT within a large general population sample. Whilst two recent reviews have shown promise for the effects of slow breathwork, they have also highlighted the risk of bias in studies to date^16^ and, in particular, the limited conclusions that can be drawn from studies using no-intervention controls^12^. In line with this, the appropriate breathwork placebo used here (paced breathing at a spontaneous frequency) may help untangle effects of CB. The primary question that our study attempted to address was: Does CB lead to improved stress (primary outcome) in comparison to an active placebo control in a general population adult sample at 4-weeks post-randomisation (post-intervention—primary timepoint)?

Fundamentally, we also set out to design a high-quality placebo for RCTs using CB which allows controlling for qualities such as attention, credibility and expectation. Since we were studying breathwork, we could manipulate the key variable of breathing pace, whilst keeping the remaining variables (i.e., duration, instruction) constant. Our intervention and placebo audio guides were identical (same background sound, and guidance to pace breathing), except for the speed at which participants were instructed to breathe. Accordingly, another important outcome from this study was whether we were able to create a much-needed placebo, which is not experienced differently from CB on credibility and expectancy (as well as being matched for duration and adherence), therefore potentially isolating the specific active ingredient of CB pertaining to this technique for future RCT research on mental health outcomes. This also enabled participant blinding to intervention allocation to be possible, very difficult to achieve in behavioural intervention studies.

### Hypotheses

For our primary hypothesis, we posited that CB for ~ 10 min/day over a 4-week period would lead to a greater reduction in self-reported (subjective) stress than practise of a breathwork placebo at post-intervention. We proposed the same at follow-up as a secondary hypothesis. Further secondary hypotheses included that CB would lead to greater improvement in subjective anxiety, depression, wellbeing and sleep disturbance than placebo at the same timepoints. As exploratory hypotheses, we postulated that greater self-reported adherence (in the intervention arm) and credibility/expectancy would be associated with greater improvements on the outcome measures at post-intervention.

## Method

### Trial design and participants

This parallel superiority RCT with 1:1 allocation was conducted online via the research platform Prolific (prolific.co). Self-assessed inclusion criteria were: Being 18 + (automatic minimum age on Prolific), able to nasal breathe, and having access to headphones. Additional pre-screeners were also set: Living in UK, English fluency, 98% + approval rate on Prolific and 20 + previous Prolific submissions (recommended by the platform to increase retention in longitudinal/multi-part studies). Exclusion (self-assessed) criteria included: problems which affected one’s ability to pace their breathing (i.e., active/chronic infection), breathlessness, cardiovascular problems, respiratory conditions or diseases (i.e., chronic obstructive pulmonary disease), abnormally slow breathing (bradypnea) or fast breathing (tachypnoea), and any other health conditions or current life events which impaired one’s ability to engage in activities involving breath control. Building on our recent meta-analysis which found a significant small-medium between-group post-intervention effect (*g* = 0.35) of breathwork on stress (primary outcome), a sample size of 260 participants was required based on an alpha level, statistical power, and estimated standardised effect size of *p* < 0.05, 0.80, and 0.35, respectively. We aimed to recruit 400 participants to allow for potential attrition.

### Randomisation and masking

Participants were enrolled through Prolific, which was integrated with the survey software Qualtrics (qualtrics.com). After completing the pre-intervention survey, participants were automatically randomly assigned (1:1) using Qualtrics via block randomisation, to receive either the intervention CB (~ 5.5 bcpm) or placebo CB (12 bcpm). The research team were blinded to randomisation but not to assignment for data analysis, due to limited time and study resources. However, participants were blinded to the study hypothesis and to their allocated intervention. The breathwork technique was referred to as ‘rhythmic breathing’ across both groups, in an attempt to conceal the specific practice being tested.

### Intervention

The intervention group received CB at ~ 5.5 bcpm (with equal inhale/exhale durations of ~ 5.5 s each), pre-recorded and guided by a trained breathwork facilitator from the organisation Othership (othership.us). Participants were asked to practise their randomly allocated breathwork session daily for four weeks: ~ 10 min/day for 28 days. The average cadence for teaching coherent breathing in the general population is around 5–6 bcpm, hence 5.5 bcpm was chosen for the intervention. The duration of ~ 10 min was deemed as a manageable time for participants, and several studies (ranging from days to weeks) on meditation have suggested benefit can be derived from this short length of practise^17–19^. Moreover, a recent remote RCT found both breathwork and meditation for as low as ~ 5 min/day significantly improved mood and state anxiety outcomes^20^.

At the start of the breathwork sessions, to get participants ready for the paced breathing, they were instructed to sit up straight or lie down in a quiet place with no disturbances, eyes closed or gaze lowered with headphones on. They were then instructed to soften their face, neck and shoulder muscles and take two deep breaths into the abdomen/diaphragm and let each one go with a sigh, softening the whole body. In order to control for as much variation as possible among participants’ respiration patterns and styles, throughout the paced breathing they were instructed to breathe softly and gently through the nose and abdominally/diaphragmatically in a balanced, rhythmic way. At the end, participants were instructed to resume normal breathing and take a moment to tune into how they felt. No loaded language pertaining to the speed of the breathing was used, nor how participants were supposed to experience the breathwork.

### Comparator

The placebo control was matched to the active intervention in all domains but pace of breathing. This group received attention-placebo CB at 12 bcpm (with equal inhale/exhale durations of 2.5 s each) recorded by the same breathwork facilitator as for the intervention. This metric was chosen in line with guidance from the Royal College of Physicians, British Journal of Nursing and Johns Hopkins University which state that the average, healthy bcpm ranges from: 12–20, 12–18 and 12–16 at rest, respectively^21–23^. Accordingly, we chose the lower bound of typical resting respiration rate in adults, since this minimum of 12 bcpm was highly unlikely to be difficult or detrimental to anyone based on such medical guidance.

### Procedure

Delivery was remotely through private audio links. Both groups were also provided with an identical ~ 5 min study introductory/welcome audio, to listen to on the first day. Participants were sent daily reminders via Prolific to practise their breathwork and keep a record of whether they had practised each day, along with reminders to complete the online surveys when necessary. Informed consent was obtained from all participants included in the study, and they were paid to complete the surveys at the Prolific-recommended rate of 9GBP/hour. All participant data were anonymous (only Prolific user IDs seen). The primary outcome was subjective stress (see below) and primary timepoint was post-intervention. Levels of stress were measured pre-post intervention and follow-up (immediately before the intervention, immediately after, and four weeks after the intervention). Secondary outcomes of subjective anxiety, depressive symptoms, wellbeing and sleep disturbance were also measured at the same timepoints (pre-post-follow-up). In addition, after the first session of practising breathwork, self-reported credibility and expectancy of the breathwork protocols for both groups were measured. Finally, self-reported adherence to the breathwork was measured at post-intervention.

### Outcome measures

Levels of stress were measured using the Depression Anxiety Stress Scale-21 (DASS-21) stress subscale (7 items)^24^. This has a response frame of the past week and score range of 0–21 with higher scores denoting worse outcomes (in line with scoring recommendations, scores are multiped by two to convert them to the longer form DASS-42 final score). For example, item 12 reads “I found it difficult to relax” and is scored from 0 (“Did not apply to me at all”) to 3 (“Applied to me very much or most of the time”). Moderate stress is scored as 19–25. Secondary outcomes of anxiety and depressive symptoms were also measured using their respective 7-item subscales. Example items for anxiety and depression include: “I was aware of dryness of my mouth” and “I couldn’t seem to experience any positive feeling at all”, respectively. The DASS has demonstrated robust psychometric properties^25–27^. At baseline, Cronbach’s alphas (*α*) revealed high internal consistency for all three subscales of DASS-21: stress (7 items; *α* = 0.86), anxiety (7 items; *α* = 0.81) and depression (7 items; *α* = 0.92).

Wellbeing was measured via the World Health Organisation-5 Well-Being Index (WHO-5)^28^. This has a response frame of the last two weeks and score range of 0–25, with higher scores denoting a better outcome. For instance, item 5 reads “My daily life has been filled with things that interest me” and is scored from 5 (“All of the time”) to 0 (“At no time”). The WHO-5 has been shown to be reliable^29,30^ and, at baseline, Cronbach’s alpha (*α*) revealed high internal consistency for this scale (5 items; *α* = 0.89).

Sleep disturbance was measured using the PROMIS Item Bank v1.0—Sleep Disturbance—Short Form 8a scale (PROMIS-8a)^31^. The PROMIS-8a has a response frame of the past week and raw score range of 5–40, with higher scores denoting a worse outcome. Moreover, data are scored using a T-score transformation in accordance with PROMIS Sleep manual guidelines (possible score range of 30.5–77.5). As an example, item 2 reads “My sleep was refreshing” and is scored from 1 (“Not at all”) to 5 (“Very much”). This PROMIS measure has well-validated items^31,32^, and Cronbach’s alpha (*α*) revealed high internal consistency for PROMIS-8a (8 items; *α* = 0.91) at baseline.

Additionally, self-reported credibility and expectancy of the breathwork protocols for both groups were measured by the Credibility/Expectancy Questionnaire (CEQ-6)^33^. The CEQ-6 has two sets of questions (credibility of course/therapy [breathwork] and expectancy of course/therapy [breathwork]). Four items are scored 1–9 and two items 0–100%. Higher scores denote greater credibility and expectancy of the course/therapy involved (in our case: CB or placebo breathwork). Example items for credibility and expectancy include “At this point, how successful do you think this breathwork will be in supporting your mental health & wellbeing?” and “By the end of the breathwork period, how much improvement in your mental health & wellbeing do you really feel will occur?”, respectively. In line with scoring guidelines for the expectancy set comprising both 1–9 and 0–100% scales, the ratings are standardised via converting them to *z*-scores. The CEQ is reliable and psychometrically well-validated^33,34^. Internal consistency of the CEQ was high for both credibility (3 items; *α* = 0.86) and expectancy (3 items; *α* = 0.89).

The final secondary outcome was self-reported adherence to the breathwork protocol (number of sessions participants reported practising out of the 28 days assigned)—measured post-intervention. Other outcomes included brief self-reports on practice impairment (i.e., whether anything hindered participants ability to perform breathwork), overall experience (i.e., positive, negative, neutral) and hypothesis guessing (to garner indication of how well blinding worked). These were measured via short optional open-ended questions to compliment the primary and secondary outcomes.

### Statistical analysis

Anonymised data were collected through Qualtrics. Data analyses were performed in *R* (version 4.1.2)^35^ using an alpha level of *p* < 0.05, with post-intervention being the primary timepoint for the primary outcome of stress. For both the primary outcome and secondary scale outcomes, group by time effects were determined using mixed repeated-measures analysis of variance. Baseline data were controlled for depending on the outcome being tested (i.e., DASS Stress at baseline was entered as a covariate when testing effects on DASS Stress). It was planned that any group by time effects at the *p* < 0.05 level would be followed up with simple contrasts (with baseline as the comparator) and Bonferroni-corrected within-group t-tests. Cronbach’s alphas (*α*) were run to inspect internal reliability of the scales at baseline. Per-protocol (participants completing at least 14 sessions, or 50%, of the breathwork practice) was conducted as the secondary analysis. Additionally, CEQ scores were compared between groups via independent t-test and correlated with the changes in pre-post outcome scale scores, adjusting alpha to *p* < 0.01 to take account of multiple correlational analyses. Self-reported adherence to the breathwork protocol was also compared between groups via independent t-test and correlated with the same changes in pre-post outcome scale scores, again adjusting alpha to *p* < 0.01 to account for multiple correlational analyses.

### Ethics and funding

The trial was approved by the Sciences and Technology Cross-Schools Research Ethics Committee (SCITEC C-REC) at the University of Sussex and pre-registered with ClinicalTrials.gov: NCT05676658 (09/01/2023). All methods used in this study were in accordance with the tenets of the Declaration of Helsinki, and all participants provided informed consent. The funder (The Ryoichi Sasakawa Young Leaders Fellowship Fund, Tokyo) played no role in the design of the trial, nor collection, analysis and interpretation of the data, along with writing up for publication.

## Results

**Figure 1:**
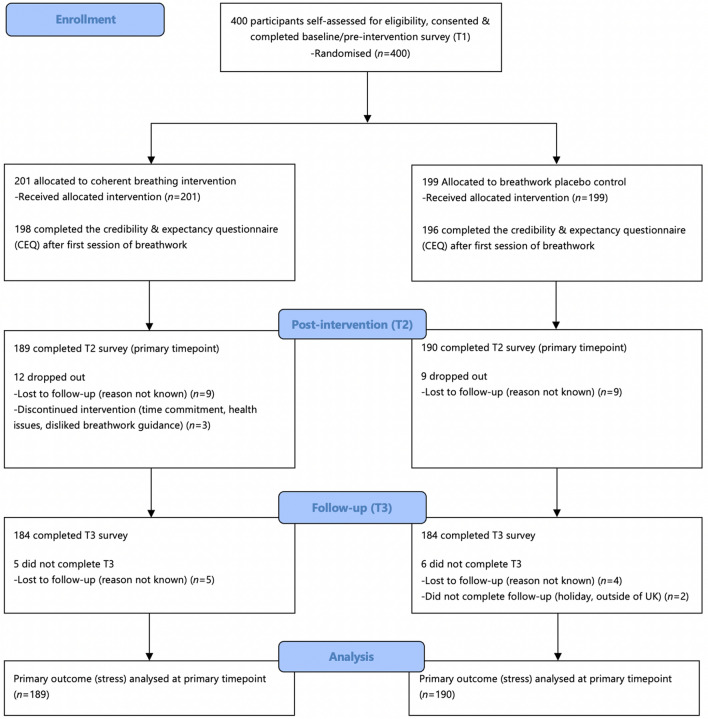
CONSORT flow diagram.

Recruitment on Prolific started and was completed on February 22. Figure 1 shows the participant flow, and baseline demographic and clinical characteristics for each group are displayed in Table 1. There were no significant differences at baseline between groups on any of these variables. More than half of participants identified as female, and most were of White ethnicity. Mean ± standard deviation (M ± SD) scores are displayed for the scales in Table 1. In addition, Table 2 shows the scores for the primary and secondary outcome measures at each timepoint.

**Table 1:** Baseline demographic and clinical characteristics for each group.

Characteristic	Group		
	Intervention (*n* = 201)	Placebo (*n* = 199)	Test statistic (*p*)
Gender (*n*)			*χ*^2^_3_ = 2.30 (0.512)
Female	125 (62.2%)	129 (64.8%)	
Male	75	69	
Other*	1	1	
Age (± SD)	39.1 ± 12.3	38.6 ± 11.0	*t* = 0.405 (0.685)
Ethnicity (*n*)			*χ*^2^_5_ = 9.50 (0.091)
White	171 (85.1%)	184 (92.5%)	
Black	11	4	
Asian	13	4	
Mixed	5	5	
Other**	1	2	
DASS-21 (M ± SD)	37.5 ± 22.7	36.5 ± 21.0	*t* = 0.468 (0.641)
Stress	15.7 ± 7.99	15.6 ± 7.77	*t* = 0.093 (0.926)
Anxiety	8.92 ± 7.12	8.55 ± 6.57	*t* = 0.530 (0.597)
Depression	12.9 ± 10.0	12.3 ± 9.36	*t* = 0.602 (0.548)
WHO-5	11.1 ± 5.27	11.6 ± 4.92	*t* = 0.930 (0.353)
PROMIS-8a	52.2 ± 3.80	52.2 ± 3.94	*t* = 0.300 (0.764)

*%* percentage within condition, *Non-binary or prefer not to say, **Any other ethnic group or prefer not to say, Mixed = 2 or more ethnic groups.

**Table 2:** Baseline, post-intervention and follow-up scores for subjective stress, anxiety and depression, along with wellbeing and sleep disturbance.

	Baseline (T1)			Post-intervention (T2)			Follow-up (T3)		
	*n*	Mean (SD)	Median (min–max)	*n*	Mean (SD)	Median (min–max)	*n*	Mean (SD)	Median (min–max)
Stress									
Intervention	201	15.7 (7.99)	16.0 (0–38)	189	12.7 (7.82)	12.0 (0–38)	184	12.1 (7.94)	12.0 (0–42)
Placebo	199	15.6 (7.77)	16.0 (0–36)	190	12.6 (7.74)	12.0 (0–40)	184	12.7 (8.25)	12.0 (0–42)
Anxiety									
Intervention	201	8.92 (7.12)	8.00 (0–28)	189	6.62 (6.66)	4.00 (0–32)	184	6.04 (6.02)	4.00 (0–26)
Placebo	199	8.55 (6.57)	6.00 (0–30)	190	5.94 (5.80)	4.00 (0–20)	184	6.41 (6.52)	4.00 (0–26)
Depression									
Intervention	201	12.9 (10.0)	10.0 (0–38)	189	9.78 (9.00)	8.00 (0–40)	184	9.76 (9.71)	6.00 (0–42)
Placebo	199	12.3 (9.36)	12.0 (0–42)	190	9.27 (8.24)	8.00 (0–42)	184	9.47 (9.68)	6.00 (0–42)
Wellbeing									
Intervention	201	11.1 (5.27)	10.0 (0–23)	189	12.9 (5.16)	14.0 (1–24)	184	12.8 (5.40)	14.0 (0–25)
Placebo	199	11.6 (4.92)	12.0 (0–21)	190	13.1 (4.98)	13.0 (2–24)	184	13.7 (5.21)	14.0 (2–25)
Sleep									
Intervention	201	52.3 (3.79)	52.3 (43–62.5)	189	52.0 (3.85)	52.0 (38.7–68)	184	52.3 (3.89)	52.4 (35.4–61.6)
Placebo	199	52.2 (3.94)	52.4 (39.2–63)	190	51.9 (3.52)	52.0 (40.7–62.5)	184	51.9 (3.26)	52.0 (42.9–62.5)

### Credibility and expectancy of breathwork protocol

394 participants completed the CEQ-6 after practising their first session of breathwork. Independent t-tests revealed no significant differences on credibility scores (M ± SD) between the intervention (17.7 ± 4.67) and control (17.7 ± 4.79), (*t*(392) = 0.124, *p* = 0.902, *d* = 0.012), nor any significant differences on expectancy *z*-scores (M ± SD) between the intervention (− 0.027 ± 1.00) and control group (0.028 ± 0.872), (*t*(392) = − 0.577, *p* = 0.564, *d* = − 0.058) suggesting no between-group differences in both expectancy and credibility. Adjusting alpha to *p* < 0.01 to take account of multiple correlational analyses, there were no significant correlations between credibility nor expectancy and pre-post intervention changes in the primary and secondary outcome scale scores.

In the intervention and control, 187 and 189 participants answered the brief optional open-ended question regarding overall experience at post-intervention, respectively. Of these responses in the CB and placebo group, 143 (76%) and 141 (75%) comprised positive sentiment, 11 (6%) and 14 (7%) negative, along with 33 (18%) and 34 (18%) neutral, respectively. The breathwork practices across both groups were generally well-received, mostly pertaining to relaxation effects (even if short-term). On top of this, no participants guessed the main hypothesis question correctly at follow-up: 80 (42%) and 75 participants (39%) answered this optional question in the intervention and control group, respectively. None mentioned the use of breathing at 5–6 bcpm and/or CB (and any of its other names, i.e., ‘resonance breathing’) nor placebo. Thus, the lack of difference on the CEQs, similar overall positive sentiment between arms, paired with blinding to the main study hypothesis, tentatively suggests masking and concealment of the intervention was successful.

### Study non-completer data and self-reported adherence

Baseline data for study non-completers, i.e., participants who did not complete post-intervention (primary timepoint) measures, were examined. There were no significant differences between study non-completers and completers (those who completed the primary timepoint) across all measures at baseline. At post-intervention, the number of participants who self-reported practising 14 or more sessions (50% +) of the 28 days assigned was 160 (85%) for the intervention group and 167 (88%) for the placebo control. There were no significant differences in self-reported number of sessions completed (M ± SDs) between intervention (20.2 ± 6.54) and control (20.5 ± 6.34) groups, (*t*(376) = − 0.434, *p* = 0.665, *d* = − 0.045). There were no significant correlations between self-reported adherence and changes in the primary and secondary outcome scale scores. In the CB group and placebo group, 59 (31%) and 47 participants (25%) reported their breathwork practice being impaired at post-intervention, respectively, the main reason being illness (namely respiratory infection) or general life circumstances (i.e., busy schedule and forgetting).

### Primary outcome: stress

**Figure 2:**
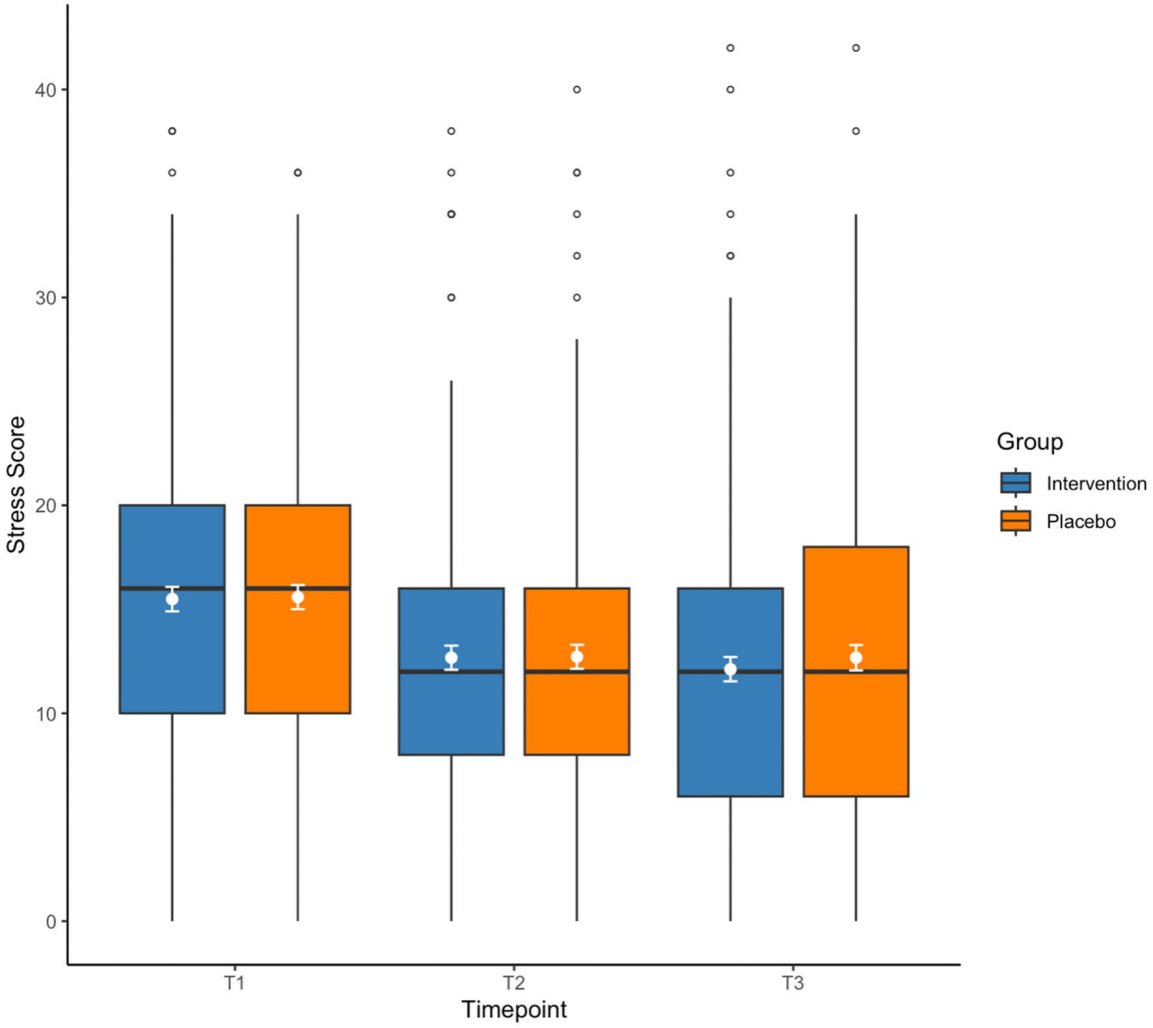
Distribution of primary outcome DASS-21 stress scores between baseline (T1), primary timepoint post-intervention (T2), and follow-up (T3) for coherent breathing (blue) and placebo breathwork (orange) groups. White dots are mean DASS stress scores for each group at each timepoint, and white error bars represent 95% confidence intervals (*CI*s). Lower scores indicate reduced stress levels, with a possible score range of 0–42. Boxes show median values (black middle lines), upper (75th) and lower (25th) percentiles; whiskers denote values within 1.5 × the interquartile range (IQR), and small empty circles are datapoints which fall outside IQR. Figure produced using *R* v4.1.2.

Regarding the primary timepoint (T2) for the primary outcome stress, there was no significant group by time interaction, (*F*(1,377) = 0.089, *p* = 0.765, *η*_*p*_^2^ < 0.001). There was no significant main effect of group, (*F* = 0.002, *p* = 0.961, *η*_*p*_^2^ < 0.001), however there was a significant main effect of time, (*F* = 72.1, *p* < 0.001, *η*_*p*_^2^ = 0.161). This shows that there was an overall improvement in stress scores from baseline to post-intervention across both groups, yet this improvement was not different between groups (see Table 2 and Fig. 2).

Furthermore, when analysing all three timepoints (T1, T2 and T3), there was no significant group by time interaction, (*F*(1,366) = 0.376, *p* = 0.540, *η*_*p*_^2^ = 0.001). Again, there was no significant main effect of group, but there was a significant main effect of time, (*F* = 0.369, *p* = 0.544, *η*_*p*_^2^ = 0.001; *F* = 71.2, *p* < 0.001, *η*_*p*_^2^ = 0.163, respectively). This shows that self-reported stress decreased over time across groups but there was no between-group difference in changes in stress (Fig. 2). Additionally, we ran a sensitivity analysis by imputing the small amount of missing data at post-intervention along with follow-up (multiple imputation of 30 datasets) and, consistent with the unimputed data, there were no significant group by time effects. A per-protocol analysis for participants who reported completing their allocated intervention (i.e., practising at least on 50% of days) replicated these findings.

### Secondary outcomes

The online supplementary material provides the mean (± 95% *CI*) scores for the secondary outcome scales at pre-post-follow-up.

### Anxiety, depression and wellbeing

For the anxiety, depression and wellbeing outcomes across the three timepoints, there were no significant group by time interactions, (*F*(1,366) = 1.12, *p* = 0.291, *η*_*p*_^2^ = 0.003; *F* = 0.007, *p* = 0.933, *η*_*p*_^2^ < 0.001; *F* = 0.024, *p* = 0.878, *η*_*p*_^2^ < 0.001, respectively). There were no significant main effects of group, (*F* = 0.495, *p* = 0.482, *η*_*p*_^2^ = 0.001; *F* = 0.627, *p* = 0.429, *η*_*p*_^2^ < 0.001; *F* = 5.45, *p* = 0.02, *η*_*p*_^2^ = 0.015, respectively). The latter *p*-value exceeded the Bonferroni corrected significance threshold. However, there were significant main effects of time, (*F* = 52.1, *p* < 0.001, *η*_*p*_^2^ = 0.125; *F* = 50.9, *p* < 0.001, *η*_*p*_^2^ = 0.122; *F* = 40.8, *p* < 0.001, *η*_*p*_^2^ = 0.100, respectively). Scores on anxiety and depression significantly decreased, while wellbeing scores significantly increased, across both groups from baseline (Table 2; Supplementary Figs. S3–S5). These findings remained for the per-protocol analysis.

### Sleep disturbance

For sleep disturbance across the three timepoints, there was no significant group by time interaction, (*F*(1,366) = 0.363, *p* = 0.547, *η*_*p*_^2^ < 0.001). There were no significant main effects of group nor time, (*F* = 1.48, *p* = 0.224, *η*_*p*_^2^ = 0.004; *F* = 0.186, *p* = 0.666, *η*_*p*_^2^ < 0.001, respectively); sleep disturbance scores were similar across all timepoints in both groups (Table 2; Supplementary Fig. S6). This also remained the case in the per-protocol analysis.

## Discussion

### Summary of findings

In the largest, blinded breathwork RCT completed to date, we compared the effects on stress, mental health, sleep and wellbeing of a coherent breathing intervention (~ 5.5 bcpm) and a well-designed placebo (matched guidance at 12 bcpm), for ~ 10 min/day over four weeks, in a general population sample. This current study did not support the main hypothesis that CB would be more effective at reducing subjective stress (primary outcome) than the placebo at post-intervention (primary timepoint). In addition to stress at the 4-week follow-up, our secondary hypotheses that CB would lead to greater improvement than the placebo on measures of subjective anxiety, depression, wellbeing and sleep disturbance, were also not supported. The most parsimonious explanation is that our results suggest CB, as it was delivered here, was simply not more effective than the breathwork placebo at reducing stress and improving mental health and wellbeing. Both breathwork groups showed significant improvement on all the mental health and wellbeing outcomes over time (except sleep disturbance—no changes). However, most importantly, there were no differences between arms on the magnitude of these improvements.

### Findings in context

Our findings are surprising in the context of a broad theoretical and empirical literature pointing to beneficial effects of slow-paced breathwork on stress, mental health, sleep and wellbeing. Recent reviews have reported on the psychological^6,8, 12^ and physiological benefits^7^ of slow-paced breathing including CB, although the methodological limitations of the field are also acknowledged. RCT studies reporting significant effects of CB on mental health outcomes and wellbeing have typically offered CB in the context of in-person training and complex intervention strategies^36–40^ and it may be the case that CB alone does not afford such benefits. When CB has been offered as an isolated intervention, relevant effects in comparison to only inactive controls have been measured and benefits may be interpreted in terms of expectation and attention^36^. One possibility is that when a more robust study design, including a well-matched placebo, is applied CB does not live up to these expectations. Further component analyses of CB like the current study, with well-matched controls, are thus needed to tease out effects, if any.

Regarding sleep outcomes, Laborde et al.’s study with 64 participants found that slow-paced breathing at 6 bcpm using a mobile application for 15 min/day before sleep over one month led to improvements in vagal activity overnight and subjective sleep quality^14^. However, as opposed to an inactive or waitlist, the control here was social media use at bedtime (to imitate archetypal smartphone use with natural respiration), which may actually be deleterious to sleep quality. Thus, it is possible that what we are actually seeing here is that slow breathwork is better than engaging in unhelpful night-time behaviour, but not necessarily more effective than spontaneous (or spontaneous rate paced) breathing without phone usage, for sleep quality.

Our results are not unique in finding null effects for CB. Several recent studies reporting on relatively brief, remote breathwork practices have found an absence of effect on similar measured outcomes. For example, an RCT comprising 80 young adults with elevated stress found no significant differences between a CB group (20 min/day, five times per week, for 28 days) and a waitlist control on subjective stress, anxiety and depression at post-intervention^41^. Alberts et al.’s remote RCT of 65 childhood cancer survivors found a significantly greater improvement on negative affect for those who completed a brief ~ 5–8 min/day mobile application breathing practice for one month compared to a waitlist, but not for positive effect, perceived stress and anxiety, nor sleep disturbance^42^. And finally, Balban et al. report on brief, remote breathwork practices finding significant effects on state affect and anxiety but no significant effects on trait anxiety or sleep-related daytime impairment outcomes^20^. However, given the small-medium, estimated effect size of breathwork interventions on psychological outcomes to date (see Fincham et al. for review^6^) none of the studies had adequate power to detect hypothesised effects, and being so, our work adds unique value to the field.

### Strengths

Firstly, this was one of the most robust, well-controlled evaluations of CB to date comprising a well-matched, credible comparator with no differences in expectation of benefit or levels of self-reported adherence. The use of a placebo such as paced respiration at a natural frequency has been posited as assisting in separating effects of CB and slow breathwork from those of purely distraction or awareness of respiration^12^. We also masked both techniques in addition to concealing the study hypotheses, a pressing challenge for behavioural intervention research like breathwork^43^. We also had very high study engagement/retention rates across both groups and thus data completeness (~ 95% for the primary timepoint of post-intervention), with the addition of a follow-up timepoint. On top of this, we had similar rates of self-reported adherence (~ 70%) to both breathwork protocols, further demonstrating the equal plausibility of the placebo. Lastly, the sample size was large enough to detect anticipated effects (*n* = 400); not only is our present study the largest RCT involving CB to date, but it is also the largest breathwork RCT in general.

### Limitations, methodological considerations and future research

Null effects may be accounted for by a number of factors. First, the intervention may have offered an inadequate dose of CB. Practices may have been too brief (in minutes), the intervention too short (in weeks), and the breathwork inadequately calibrated or insufficiently contextualised in this fully remote intervention design. Participants were offered a single, recorded ~ 10 min CB practice for daily engagement over four weeks. Self-reported engagement indicated 20 sessions per participant on average, although previous work has suggested a significant optimistic bias in reporting engagement with brief digital interventions^44^. One possibility is that this is simply not enough CB to have any measurable benefit, and future research could explore a range of breathwork doses which might identify what is needed for effects to occur. This could also be combined with the use of more ecologically valid methods to assess adherence.

Second, it may be the case that this would be an adequate dose of CB if what was practised was well-calibrated to the method. Remote non-personalised delivery meant that there was no real-time feedback, visual or otherwise, to ensure participants were performing CB in an ideal manner (i.e., abdominally/diaphragmatically, pacing breathing correctly and/or not pausing for breath). In addition, to ensure methodological rigour and successful blinding, there was no psychoeducation nor context provided for why participants were breathing at the rate of ~ 5.5 bcpm. The standardisation and unsupported nature of this online CB—without expectation-loaded language related to the exact breathing rate used and desired subjective experiences—meant that such neutrality could have diluted intention and meaning of the technique itself. In most contemplative practices including mindfulness, meditation, yoga, and pranayama (breathwork), context and learning are regularly provided before teaching a specific technique. Psychoeducation paired with breathwork could thus complement practices and lead to improved, prolonged outcomes. In summary, the full potential of CB was not adequately realised by following this standalone recorded guidance. However, such reduction in standardisation of protocols could introduce numerous confounding variables, such as effects pertaining to artificially enhancing expectation of benefit, cognition, the breathwork facilitator and individualised guidance, making findings difficult to interpret. This also decreases accessibility compared to entirely remote, self-help delivery and reduces the feasibility of recruiting a large general population sample as we successfully did here.

Third, it is possible that in creating a 12 bcpm paced, abdominal/diaphragmatic, nasal breathwork of this kind we have inadvertently developed an active intervention of equivalent potency to CB at this small dose. Indeed, in addition to the lack of difference on the credibility and expectancy measures, there was similar overall positive sentiment between arms in qualitative feedback. Thus, our findings could reflect the effects of two active intervention arms rather than one intervention versus a placebo. Whilst intended to reflect a spontaneous breathing rate it is possible that paced breathing alone (as opposed to un-paced) has some beneficial effects. Perhaps simply ‘conscious breathing’ that invites attention to the breath, and regulates breathing pace, without significantly slowing it can improve stress. It is also likely that the placebo breathing pace was in the lower range of average resting respiration rate for most participants, and therefore could be considered a ‘slower’ breathwork, in turn masking the effects of CB in our study. There is little available evidence that paced breathing in the range of 12 bcpm is associated with significant benefits of this kind. However, one study with 53 healthy participants found a rise in vagal activity and shift towards parasympathetic nervous system balance (i.e., the ‘rest and digest’ response) when breathing at a rate of 8 bcpm and 12 bcpm compared to 16 bcpm, though results yielded for 8 bcpm were more pronounced and significant overall^45^. Future slow-paced breathwork research could develop an equally plausible, but less potentially active comparator that is either slightly more rapid (15–16 bcpm) or tailored to the participants’ spontaneous respiration frequency (or un-paced), not specifically inviting abdominal/diaphragmatic or nasal breathing.

Pace aside, in our study it is possible that the paced breathing guidance in both groups led to beneficial deeper breathing, and abdominal/diaphragmatic breathing alone could be responsible for the change in outcomes in both conditions. Participants in both groups were instructed to breathe abdominally/diaphragmatically and through the nose, a method which has been shown to be associated with decreases in DASS stress, anxiety and depression in another study where participants practised just ~ 5/min daily diaphragmatic breathing exercises (inhale through nose and controlled exhale through mouth at an unspecified rate) followed by ~ 5 min of rest, over one month, compared to standard care^46^. However, these participants received significantly more guidance and training than those in the current study, and were initially taught by the research team in-person to ensure proper adherence to the breathwork protocol.

Alternatively, nasal breathing was also invited in all participants and could also be responsible for change in both conditions. In comparison to mouth breathing, nasal breathing can result in 10–20% greater oxygen intake^12^ and affects certain brain regions involved in emotion regulation differently, which has treatment implications for stress management and anxiety^20^. It has been shown that in nasally obstructed symptomatic patients, those with regular nasal breathing reported significantly better mental health along with physical quality of life outcomes, in comparison to those who had compromised nasal breathing^47^. Therefore it is possible that nasal breathing may confer benefits to these outcomes with practise, but more research is needed to confirm this^8,12^. Zaccaro et al.^8^ suggest that nasal stimulation underpins the essential connection between slow breathwork and therapeutic psychophysiological outcomes, including altered states of consciousness phenomenologically corresponding to those induced by intensive meditation^48^. Indeed, the modulating impact of nostril breathing in humans on activity of certain brain regions involved in the processing of memory, emotion and behaviour (i.e., piriform cortex, hippocampus and amygdala) has been firmly established^49^.

So, it is possible that our placebo conferred specific, unanticipated benefits to participants in this study through paced, abdominal/diaphragmatic and nasal breathing instructions, masking a real benefit of CB. However, it is, at least, equally plausible that the changes over time seen in both conditions may be explained by effects of expectation, attention, history, maturation or regression to the mean^13^.

Another possibility is that beneficial effects of CB were missed by our methods. Perhaps CB is effective but not in the sample recruited to our study using the Prolific platform who may not be representative of the general population (cf. Peer et al.^50^) or representative of those who may benefit most from CB. Replication in a non-Prolific recruited sample may be warranted. One possibility is that CB may have its most potent effects in those with higher levels of stress or mental health symptoms, and there is some evidence to suggest that CB (in the context of a more complex intervention protocol) may have benefits for such populations^37–40^. In order to explore this idea, we performed a post-hoc exploratory analysis replicating our primary analysis in only those study participants scoring above the normal range for stress (15 or more) on the DASS stress subscale. There was no evidence of effect in the group by time interaction which remained non-significant with a negligible effect size, (*F*(1,188) = 0.648, *p* = 0.422, *η*_*p*_^*2*^ = 0.003), and therefore no evidence to support the idea that CB alone is more effective than a placebo for a more symptomatic sample, though further research specifically designed to test this hypothesis is needed.

Alternatively, it is possible that CBs distinct effects are more physiological than psychological, and registered in short-term cardiovascular changes rather than a shift in felt sense of stress resilience. For example, a study with inflammatory bowel disease patients showed significant psychological improvements along with reductions in C-reactive protein (a biomarker for inflammation)^37^. This suggests potential psychophysiological change as a result of CB, and in an fMRI study with 20 healthy participants, slow breathing at 5.5 bcpm was found to mitigate cardiac and autonomic stress responses to hypoxic challenge^51^. Future research exploring the psychophysiological effects of CB in well-controlled studies will further our understanding here.

It is also possible that in the case of a ‘low dose’ CB intervention of this kind, the effects may be momentary but not long-lasting. Previous work has reported beneficial effects on state mood and state anxiety, but not perceived stress, trait anxiety, depression or sleep disturbance associated with brief/remote breathwork^20, 36,42^ and therefore further research efforts (particularly regarding slow breathwork) could focus on examining state outcomes rather than trait measures insensitive to changes. Indeed, qualitative feedback from participants in both groups reported beneficial, calming and relaxation effects, with the frequent caveat that this was temporary, i.e., feeling relaxed during and after breathwork sessions, but not experiencing enduring, long-term change—which is consistent with changes in breathing pace and style having short-term physiological effects.

The question remains whether regular breathwork practise at a specific dose can extend beneficial transient effects to enduring change or allow people to access these emotional state shifts more readily and quickly. Thus, trait outcomes are still important as, akin to physical exercise, the accumulation of state benefits could lead to such durable change and improved ability to access shifts in state. While mechanisms of action for breathwork, or at least brief practices, may therefore possibly be more physiological and short-lasting (state) versus cognitive and potentially longer-term (trait), greater practise (dose) could mean practitioners can access such shifts in emotional state when desirable/needed, i.e., wanting to feel calm in real-time and thus incorporating ‘situational breathwork’ in the moment.

## Conclusion

To the best of our knowledge, we designed the largest and one of the most robust, blinded RCTs exploring coherent breathing, and breathwork in general. Both interventions were masked as rhythmic breathing (equal inhale:exhale ratios and no deliberates pauses) at a rate of ~ 5.5 bcpm (CB) versus 12 bcpm (placebo), the lower bound pace of typical resting adult respiration rate. Importantly, both breathwork practices demonstrated equal plausibility in terms of credibility and expectancy of benefit, paired with similar adherence, engagement and positive sentiment. The study suggests that remote delivery of a 4-week unsupported, blinded CB intervention of ~ 10 min/day did not have any measurable effect over and above a well-designed placebo at improving mental health and wellbeing. This is not to say CB in general does not help people—this finding may be related to our intervention approach and methods, and sentiment was largely positive towards the CB protocol (along with the placebo). In summation, CB may have effects, but not on what we measured. Perhaps the effects are more physiological than psychological, or effects may be more immediate and more transient than our measurement regime was able to capture. The intervention could have been inadequate (too brief, not done correctly, with not enough context) and the comparator could have been an inadvertent active intervention, through the action of (slow)paced, abdominal/diaphragmatic and/or nasal breathing methods.

Results such as this from a rigorous study help to calibrate hype with evidence for (slow) breathwork early on in this emerging research field’s development. Future research is needed to explore the potential for brief, unsupported CB interventions, but must also focus on developing more intensive, personalised CB interventions with equally well-matched placebos in order to categorically determine if CB itself is specifically effective or not at improving mental health and wellbeing. The same can be said for myriad breathwork techniques in general and we hope our methodological considerations raised can help future breathwork research and practice, in a time where simple and effective tools for fostering stress resilience are needed most.

### Supplementary Information

Supplementary Information.

## Data Availability

The datasets used and/or analysed during the current study available from the corresponding author on reasonable request.
