# Supplementary material for: Effect of coherent breathing on mental health and wellbeing: a randomised placebo-controlled trial
Source: Sci Rep. 2023 Dec 13;13:22141. doi: 10.1038/s41598-023-49279-8 (PMC10719279; doi:10.1038/s41598-023-49279-8)
Supplement: Supplementary file 1 — Supplementary Information. [file 41598_2023_49279_MOESM1_ESM.pdf]

Supplementary material

Effect of coherent breathing on mental health and wellbeing: A randomised placebo-controlled trial

Mr Guy William Fincham[1,2*], MSc, Prof Clara Strauss[1,3], DClinPsy, PhD, Prof Kate Cavanagh[1,3], DClinPsy, PhD

[1] Department of Psychology, University of Sussex, Brighton, UK
[2] Brighton & Sussex Medical School, University of Sussex, UK
[3] Research & Development Department, Sussex Partnership NHS Foundation Trust, UK
*Corresponding author

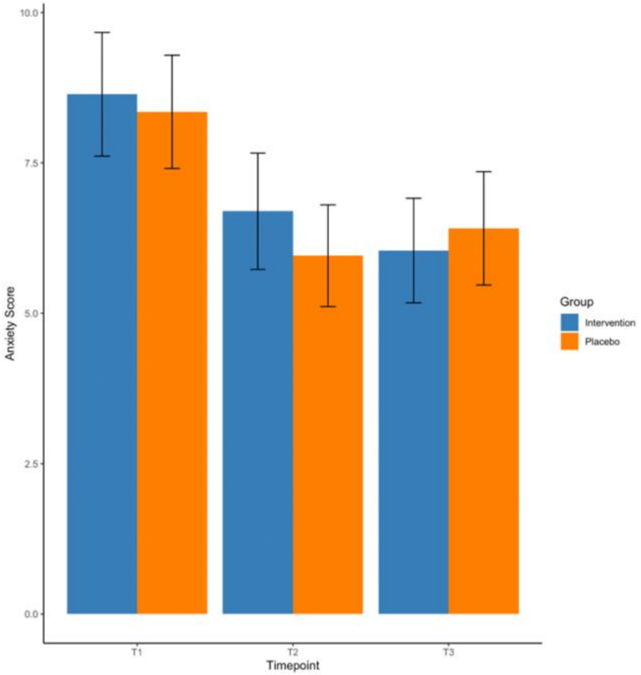

Figure 3. Mean (± 95% *CI*) DASS anxiety scores for the intervention and placebo groups at pre-post-follow-up (T1, T2, and T3). Blue bars are coherent breathing intervention group scores and orange bars are placebo breathwork group scores at each timepoint. Lower scores denote reduced anxiety levels, with a possible score range of 0-42. Figure produced using *R* v4.1.2.

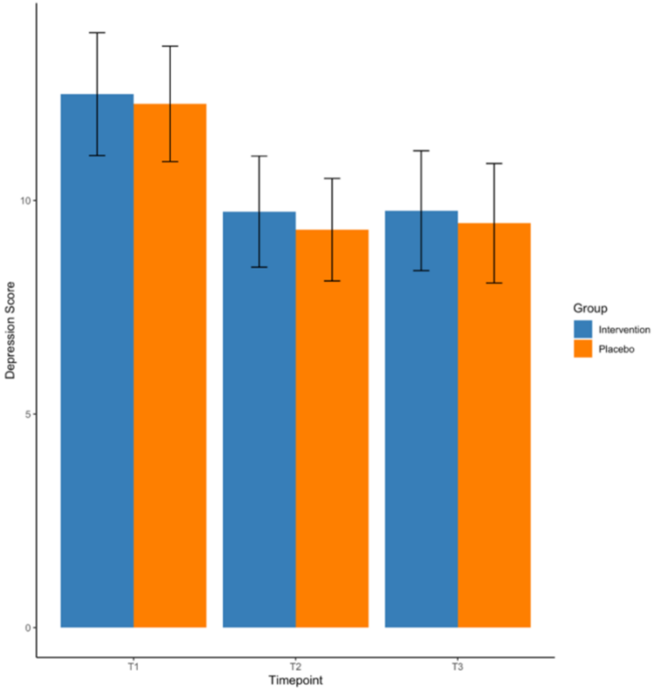

Figure 4. Mean (± 95% *CI*) DASS depression scores for the intervention and placebo groups at pre-post-follow-up (T1, T2, and T3). Blue bars are coherent breathing intervention group scores and orange bars are placebo breathwork group scores at each timepoint. Lower scores denote less depressive symptoms, with a possible score range of 0-42. Figure produced using *R* v4.1.2.

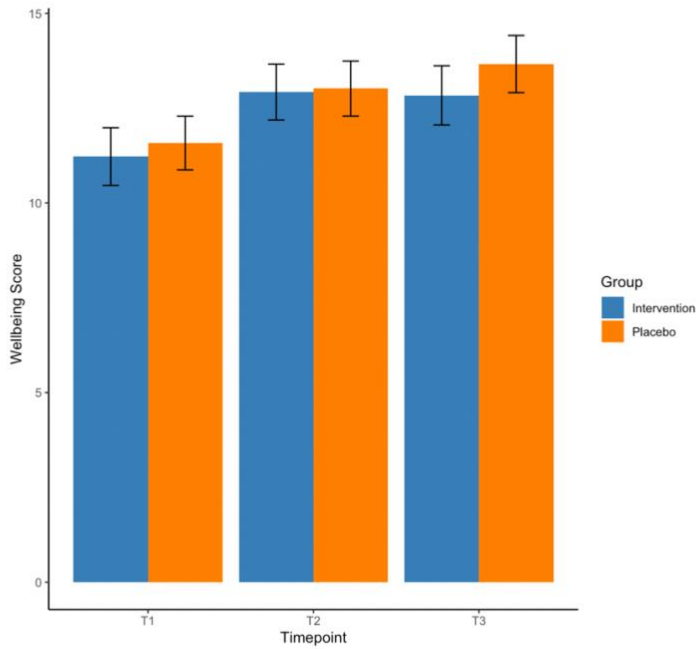

Figure 5. Mean (± 95% *CI*) WHO-5 wellbeing scores for the intervention and placebo groups at pre-post-follow-up (T1, T2, and T3). Blue bars are coherent breathing intervention group scores and orange bars are placebo breathwork group scores at each timepoint. Higher scores denote greater levels of wellbeing, with a possible score range of 0-25. Figure produced using *R* v4.1.2.

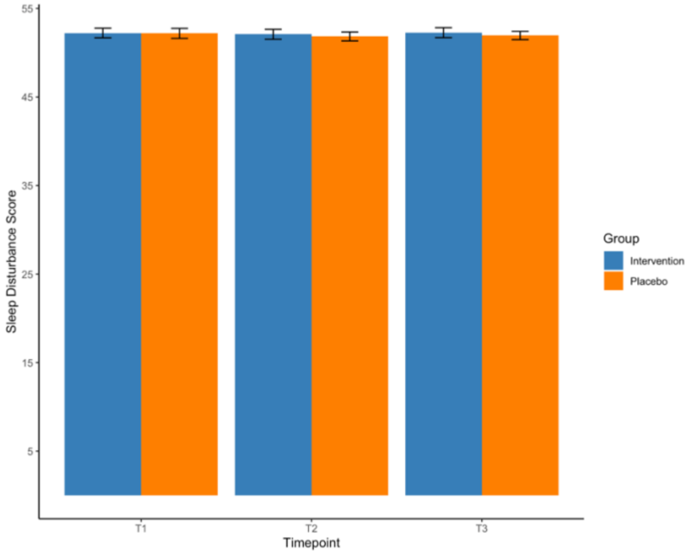

Figure 6. Mean (± 95% *CI*) PROMIS-8a sleep disturbance scores for the intervention and placebo groups at pre-post-follow-up (T1, T2, and T3). Blue bars are coherent breathing intervention group scores and orange bars are placebo breathwork group scores at each timepoint. Lower scores denote less sleep disturbance, with a possible score range of 30.5-77.5. Figure produced using *R* v4.1.2.
